# Drinking coffee enhances neurocognitive function by reorganizing brain functional connectivity

**DOI:** 10.1038/s41598-021-93849-7

**Published:** 2021-07-13

**Authors:** Hayom Kim, Sung Hoon Kang, Soon Ho Kim, Seong Hwan Kim, Jihyeon Hwang, Jae-Gyum Kim, Kyungreem Han, Jung Bin Kim

**Affiliations:** 1 Department of Neurology, Korea University Anam Hospital, Korea University College of Medicine, Seoul, Republic of Korea; 2 Department of Neurology, Samsung Medical Center, Sungkyunkwan University School of Medicine, Seoul, Republic of Korea; 3 Department of Neurology, Korea University Guro Hospital, Korea University College of Medicine, Seoul, Republic of Korea; 4 Laboratory of Computational Neurophysics, Brain Science Institute, Korea Institute of Science and Technology, Seoul, Republic of Korea

**Keywords:** Cognitive neuroscience, Computational neuroscience

## Abstract

The purpose of this study was to identify the mechanisms underlying effects of coffee on cognition in the context of brain networks. Here we investigated functional connectivity before and after drinking coffee using graph-theoretic analysis of electroencephalography (EEG). Twenty-one healthy adults voluntarily participated in this study. The resting-state EEG data and results of neuropsychological tests were consecutively acquired before and 30 min after coffee consumption. Graph analyses were performed and compared before and after coffee consumption. Correlation analyses were conducted to assess the relationship between changes in graph measures and those in cognitive function tests. Functional connectivity (FC) was reorganized toward more efficient network properties after coffee consumption. Performance in Digit Span tests and Trail Making Test Part B improved after coffee consumption, and the improved performance in executive function was correlated with changes in graph measures, reflecting a shift toward efficient network properties. The beneficial effects of coffee on cognitive function might be attributed to the reorganization of FC toward more efficient network properties. Based on our findings, the patterns of network reorganization could be used as quantitative markers to elucidate the mechanisms underlying the beneficial effects of coffee on cognition, especially executive function.

## Introduction

Coffee is a widely used caffeinated beverage (International Coffee Organization, http://www.ico.org/prices/new-consumption-table.pdf.), with more than 165 million 60-kg bags consumed globally per year^1^. Potential beneficial health effects of coffee consumption have been reported, including prevention of cancer, cardiovascular disorders, diabetes, and Parkinson's disease^2^. Furthermore, given the expectation that coffee increases alertness and enhances psychomotor functioning, many people seek coffee to counteract fatigue, stay alert by warding off sleepiness, increase cognitive performance, and increase work efficiency^3^.

The stimulatory effects of coffee are mainly attributed to caffeine’s roles in antagonizing adenosine A_1_ and A_2A_ receptors, leading to disinhibition of excitatory neurotransmitter release and enhancement of dopamine transmission via D_2_ receptor, respectively^4^. Although it is agreed that the acute effects of caffeine are due to its action as a central stimulant, inconsistent findings have been reported regarding the effects of coffee on higher cognitive functions, including working memory and executive functioning^5^. Some studies have shown beneficial effects of caffeine on cognitive functioning, including reaction times to cognitive tasks^6^, attention^7^, working memory^8^, and executive control^9,10^, whereas others reported no change after the use of caffeine^11,12^. Moreover, the expectancy of the stimulant effects of caffeine itself may have a role in the cognitive responses to caffeine^12^. Therefore, whether the beneficial effects of caffeine are derived from the direct enhancement of specific cognitive functions is unclear.

Integration of neural activities between different brain regions is required for physiological brain functioning. Therefore, analyzing functional connectivity (FC) between brain regions may provide more information than investigating activities of individual brain regions^13,14^. While most of the previous studies have evaluated the cognitive effects of caffeine mainly based on the results of neurocognitive tests, such as the Stroop test^5^, little attention has been paid to investigating the effects of caffeine on neurocognitive function in the context of FC. Recent advances in graph-theoretic network analysis allow for the assessment of important information regarding the topological architecture of complex human brain networks^15,16^. Therefore, graph-theoretic analysis could be an optimal framework for quantitatively characterizing network properties after coffee consumption and determining its effects on cognition.

To the best of our knowledge, no graph-theoretic analysis has applied electroencephalography (EEG) data to explore the effects of caffeine on FC. Here, we compared the properties of FC before and after coffee consumption to analyze the acute effects of caffeine on the brain network and its impact on neurocognitive function using graph-theoretic analysis of EEG data. We hypothesized that caffeine might improve neurocognitive function by shifting the FC of the brain to a more efficient state.

## Methods and materials

### Participants

Twenty-one healthy volunteers (11 women; 31.4 ± 3.9 years; 17.0 ± 1.4 years of education) who had no neurologic, psychiatric, chronic systemic disorders, or medical conditions that could affect the EEG results were included in this study. All participants were requested to abstain from drinking beverages containing caffeine and from the use of any psychoactive substances or medication for at least 24 h prior to the EEG and neurocognitive studies^17^. All subjects were fully informed of the nature and possible risks of this study. Written informed consent was obtained from all subjects prior to study enrollment. The study followed the ethical guidelines of the Declaration of Helsinki and was approved by the local ethics committee at Korea University Anam Hospital (No. 2019AN0418).

### Neurocognitive function tests and caffeine intake

Global neurocognitive function was assessed using the Mini-Mental State Examination (MMSE) at least 24 h after cessation of coffee consumption in all participants. The neuropsychological tests were selected to evaluate the acute effects of caffeine on performance in multiple neurocognitive domains. The assessed domains and the tests were as follows: (1) attention and working memory—Digit Span Forward (up to nine digits) and Backward (up to eight digits) tests^18^, Target Detection Task using tapping; (2) executive function—Trail Making Test Part B (time to complete the tests)^19^; and (3) memory—Short-term memory recall task (two learning trials of five words) and delayed recall (after 5 min). All neuropsychological tests were performed at baseline EEG recording and 30 min after consumption of canned coffee^19^ using the same tests with a different set of contents. A commercial canned coffee (Let’s Be, Lotte Chilsung Beverage), which has the largest market share in Korea, was used for caffeine intake. One can of the canned coffee contains 160 mL and 67 mg of caffeine.

### Electroencephalography recording

**Figure 1:**
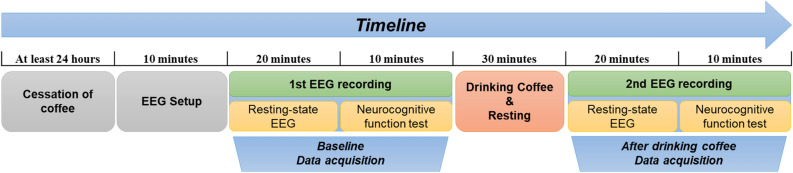
A diagram of the study protocol. The study protocol according to the timeline is presented schematically.

The EEG examination was performed twice, once at baseline and then again 30 min after the participants drank the canned coffee treatment^20^, using a 32-channel recording system (Comet-PLUS, Grass Technologies Inc., West Warwick, RI, USA) with electrodes placed according to the international 10–20 system. The EEG was recorded for 1 h in the waking-relaxed and eyes-closed conditions. EEG data were sampled at 200 Hz, and the bandpass filter was set between 0.1 and 70 Hz. A diagram of the study protocol is presented in Fig. 1.

Since the neuropsychological tests evaluating different domains were performed in succession, resting-state EEG data were used for analysis to avoid mixed effects of different domain-specific functional networks in this study. Ten non-consecutive resting-state 2-s epochs for each participant were carefully reviewed and selected by two board-certified neurologists according to the following protocol: (1) presence of continuous physiological alpha activity with voltage maximum in posterior regions; (2) absence of artifacts, epileptiform discharges, and other nonstationary elements; and (3) absence of patterns indicating drowsiness or arousal.

### Graph-theoretic and statistical analyses

Resting-state FC was evaluated by coherence, which reflects the level of functional signal communication between different regions of the brain^21^. The coherence is defined as

$$COH_{{xy}} ~ = k_{{xy}} ^{2} (f) = \left| {K_{{xy}} (f)} \right|^{2} = ~\frac{{\left| {S_{{xy}} (f)} \right|^{2} }}{{S_{{xx}} (f)S_{{yy}} (f)}},$$

where *S*_*xy*_(*f*) is the cross-spectral density between *x* and *y*, and *Sxx*(*f*) and *Syy*(*f*) are the auto-spectral densities of *x* and *y*, respectively. K represents the coherency function. |*S*| denotes the modulus of *S*. The coherence value ranges between 0 and 1 with 0 denoting no statistical relationship and 1 being full coherence^21^. In addition, the phase lag index (PLI) was used to measure phase synchronization between all pairs of 19 EEG channels^22,23^. The PLI is defined as *PLI* = |< sign[Δφ(*t*_*k*_)] >|, where Δφ(*t*_*k*_) is the phase difference of time series *t*_*k*_ (*k* = 1, …, *N*). It ranges between 0 and 1—0 indicates either no coupling or phase difference centered around 0 mod π, while 1 indicates perfect phase synchrony as a value of Δφ different from 0 mod π. Epochs were then bandpass filtered into the following frequency bands: delta (0.1–4 Hz), theta (4–8 Hz), alpha (8–13 Hz), beta (13–30 Hz), and gamma (30–50 Hz). Subsequent analyses were performed separately for each band. Network properties were characterized using a weighted undirected network model of graph-theoretic analysis in order to avoid the arbitrariness of threshold selection for producing an adjacency matrix and to preserve the continuous nature of the correlated information^24^. Graph measures (average degree, average strength, radius, diameter, characteristic path length, global and local efficiency, clustering coefficient, transitivity, modularity, assortativity, and small-worldness) were computed using the Brain Connectivity Toolbox (http://www.brain-connectivity-toolbox.net) and BRAPH toolbox (http://braph.org) working on MATLAB R2019b (MathWorks, Natick, MA, USA)^24,25^. PLI analysis and visualization were performed using tailored Python scripts and the MNE-Python package (version 0.22.0)^26^.

Graph measures were compared before and after consumption of canned coffee using non-parametric tests with 1,000 permutations. Statistical significance was set at P < 0.05 and corrected for multiple comparisons using false discovery rate (FDR). Differences in the results of neuropsychological tests before and after consumption of canned coffee were compared using paired *t*-test. Since the ranges of the graph measures’ values differed from each other, the degree of change was normalized, and then the correlation between the score changes of Trail Making Test Part B and the graph measures was analyzed using the normalized changed values (Pearson’s correlation, P < 0.05). The purpose of the statistical tests is to determine whether each of the graph measures is correlated with the score change of Trail Making Test Part B. Accordingly, the statistical test was performed independently for each graph measure with respect to the neuropsychological test.

## Results

### Neuropsychological tests

**Figure 2:**
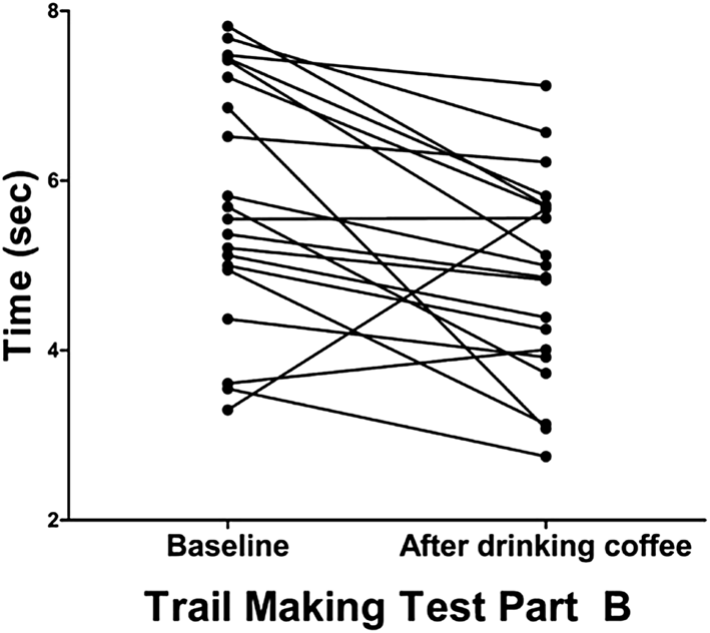
Acute effects of coffee consumption on executive function. Individual changes in time (s) to complete Trail Making Test Part B are presented.

The results of the neuropsychological tests are detailed in Table 1. All participants had an MMSE score of 30. Performance in the Digit Span Forward (8.5 ± 0.8 digits vs. 8.9 ± 0.2 digits, P = 0.025) and Backward tests (6.2 ± 1.8 digits vs. 7.3 ± 1.1 digits, P = 0.001) improved after coffee consumption relative to baseline. There were no errors in the target detection task using the tapping test, Trail Making Test Part B, and short-term and delayed memory recall tests before and after coffee consumption. Compared to baseline, performance in the Trail Making Test Part B improved after coffee consumption (5.8 ± 1.4 s vs. 4.9 ± 1.2 s, P = 0.002). Individual changes in performance in the Trail Making Test Part B after coffee drinking are presented in Fig. 2.

**Table 1:** Results of neuropsychological tests.

	Baseline	After coffee consumption	P value
**<Global function>**			
Mini-mental status examination (correct/30 items)	30	30	–
**<Attention and working memory>**			
Digit Span Test forward (correct/9 digits)	8.52 ± 0.75	8.95 ± 0.22	0.025
Digit Span Test backward (correct/8 digits)	6.24 ± 1.76	7.29 ± 1.10	0.001
Target Detection Task (correct/11 syllable targets)	11	11	–
**<Executive function>**			
Trail Making Test Part B (s)	5.80 ± 1.41	4.87 ± 1.17	0.002
**<Memory>**			
Short-term memory recall (correct/5 words)	5	5	–
Delayed recall (correct/5 words)	4.57 ± 0.68	4.81 ± 0.40	0.135

Values represent mean ± standard deviation.

### Graph-theoretic analyses

**Figure 3:**
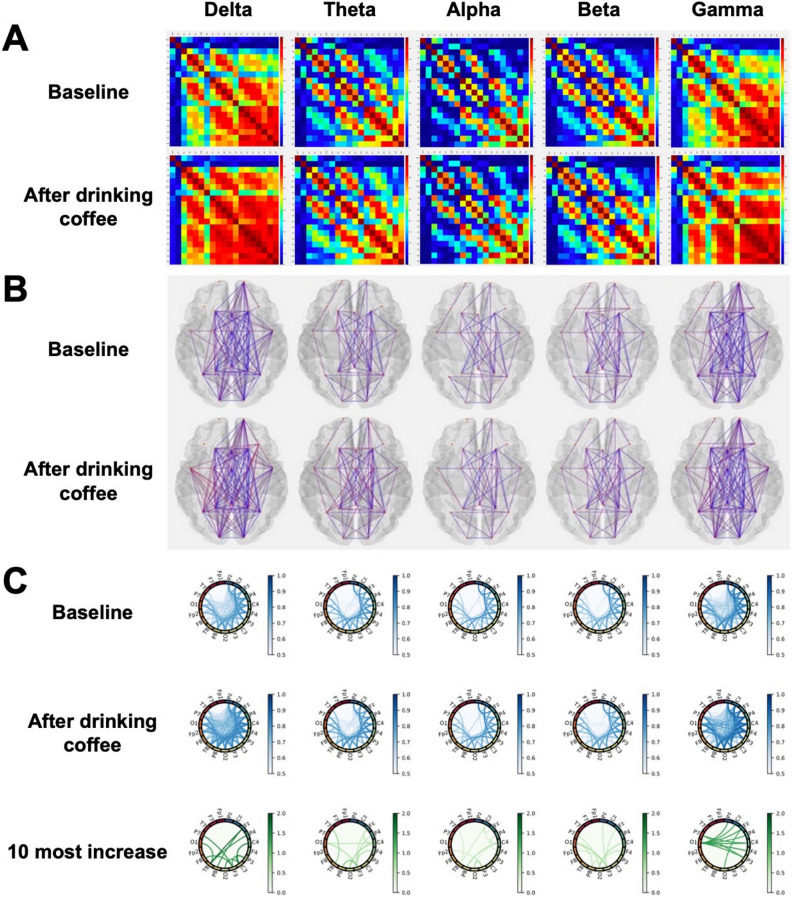
Coherence averaged across all subjects. (**A**) The plots show the coherence between 19 pairs of scalp electroencephalography electrodes in each frequency band at baseline (upper) and after coffee consumption (low). (**B**) Brain topologies of functional connectivity at baseline (upper) and after coffee consumption (low) are presented. (**C**) Each column corresponds to a frequency band as indicated on the top of the figure. Coherence matrices corresponding to the resting-state EEG before and after coffee consumption are plotted on the first and second rows, respectively; to avoid confusion from too many lines, those of coherence less than 0.5 are not shown. The third row displays the top 10 most increased lines.

**Figure 4:**
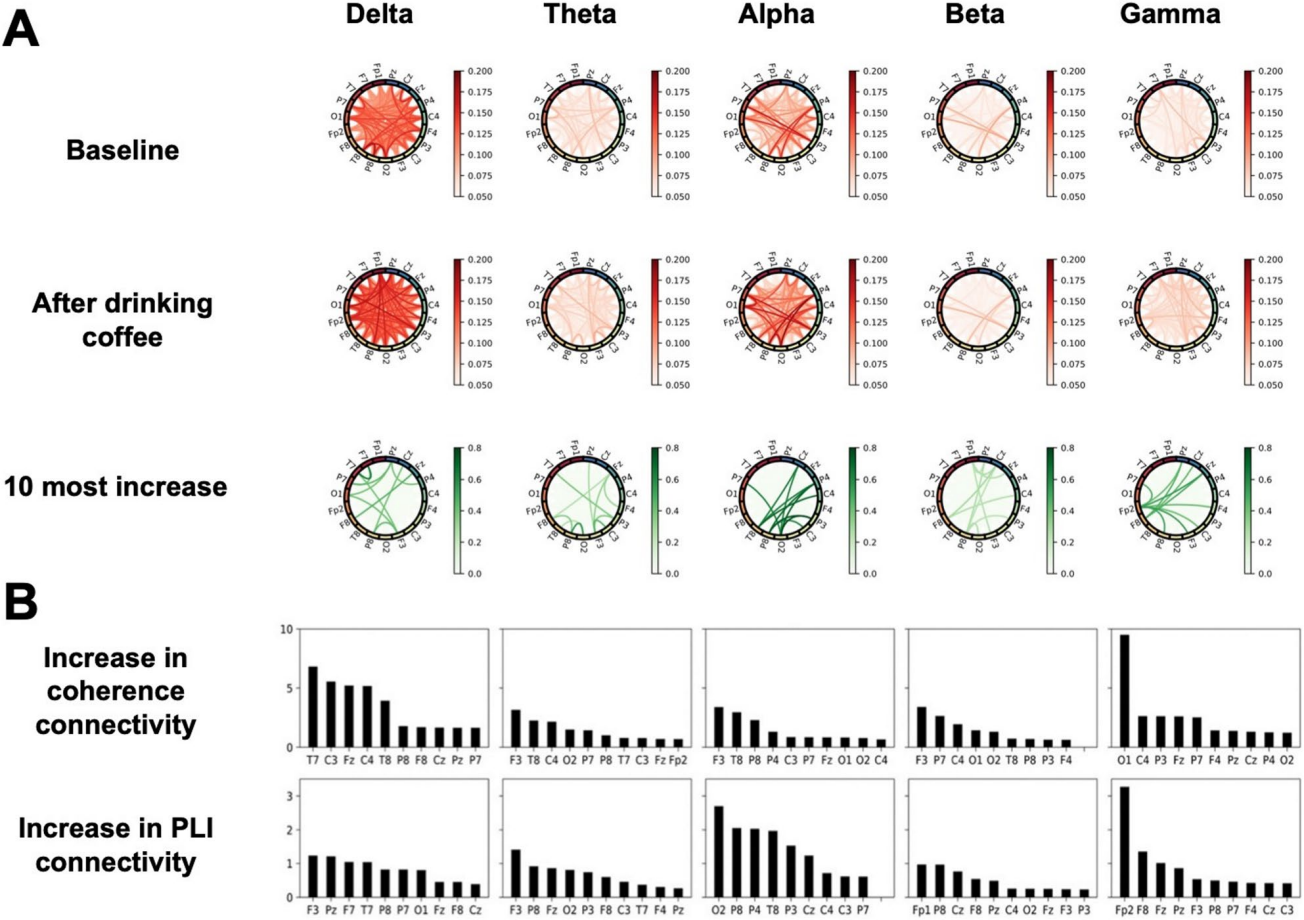
Phase lag index (PLI) averaged across all subjects and ten most highly increased nodes in each band. (**A**) Each column corresponds to a frequency band as indicated on the top of the figure. PLI matrices corresponding to the resting-state EEG before and after coffee consumption are plotted on the first and second rows, respectively; lines that have PLI values under 0.05 are not displayed. The third row plots the top 10 most increased lines. (**B**) The ten channels (nodes) with the highest increase in connectivity in terms of coherence (top row) and PLI (bottom row) are shown for each frequency band (corresponding to each column). Node connectivity was determined by taking the ten most highly increased links after coffee consumption from the averaged functional connectivity matrix (as in the third rows of Figs. 3C and A) and summing the weights of those edges connected to each node.

FC in terms of coherence is represented with adjacent matrices, connectivity circles, and brain topologies in Fig. 3A–C. PLIs are presented using the connectivity circles in Fig. 4A. FC was enhanced in all frequency bands for both methods, and similar patterns were found—the delta and gamma bands exhibited relatively large increases in both coherence (Fig. 3C) and PLI (Fig. 4A); for coherence, the theta, alpha, and beta bands increase less than those of delta and gamma; the alpha band also largely increased for PLI. The ten most highly increased connectivities are displayed in Fig. 4B; this indicates the relevant brain regions responsible for the increase of FC. Comparisons of global graph measures between the conditions are detailed in Table 2.

**Table 2:** Comparisons of global graph measures between baseline and after coffee consumption.

Graph measures	Baseline	Coffee	P value	Baseline	Coffee	P value	Baseline	Coffee	P value	Baseline	Coffee	P value	Baseline	Coffee	P value
	Delta			Theta			Alpha			Beta			Gamma		
Average degree	13.920	15.058	0.001	12.005	12.988	0.013	10.782	11.378	0.043	12.145	13.033	0.027	14.852	15.649	0.061
Average strength	9.253	11.333	0.001	6.175	7.338	0.010	5.037	5.434	0.021	5.924	6.376	0.125	9.453	10.685	0.012
Radius	5.885	6.120	0.810	13.820	6.191	0.923	5.481	5.760	0.218	7.162	5.194	0.112	4.414	4.336	0.915
Diameter	10.702	9.697	0.586	17.776	10.809	0.948	9.598	9.773	0.720	11.061	9.066	0.459	7.952	7.544	0.510
Characteristic path length	2.799	2.377	0.218	4.071	3.056	0.183	3.520	3.377	0.228	3.598	3.116	0.054	2.449	2.182	0.066
Global efficiency	0.568	0.659	0.004	0.444	0.488	0.015	0.397	0.412	0.054	0.428	0.445	0.108	0.580	0.640	0.029
Local efficiency	1.718	2.255	0.001	1.057	1.267	0.019	0.826	0.884	0.026	0.945	1.004	0.208	1.696	1.958	0.049
Clustering coefficient	0.560	0.670	0.002	0.380	0.439	0.035	0.328	0.341	0.197	0.357	0.372	0.277	0.542	0.605	0.052
Transitivity	0.913	1.083	0.002	0.595	0.699	0.022	0.500	0.525	0.140	0.554	0.581	0.208	0.858	0.951	0.046
Modularity	0.063	0.026	0.010	0.188	0.137	0.001	0.258	0.241	0.329	0.195	0.171	0.101	0.070	0.036	0.011
Assortativity	0.111	0.050	0.188	0.160	0.077	0.105	0.156	0.123	0.223	0.122	0.080	0.116	0.025	0.019	0.202
Small-worldness	1.042	1.233	0.612	0.855	1.250	0.289	0.891	1.054	0.460	0.868	0.945	0.364	0.927	0.901	0.166

Values represent the mean.

**Figure 5:**
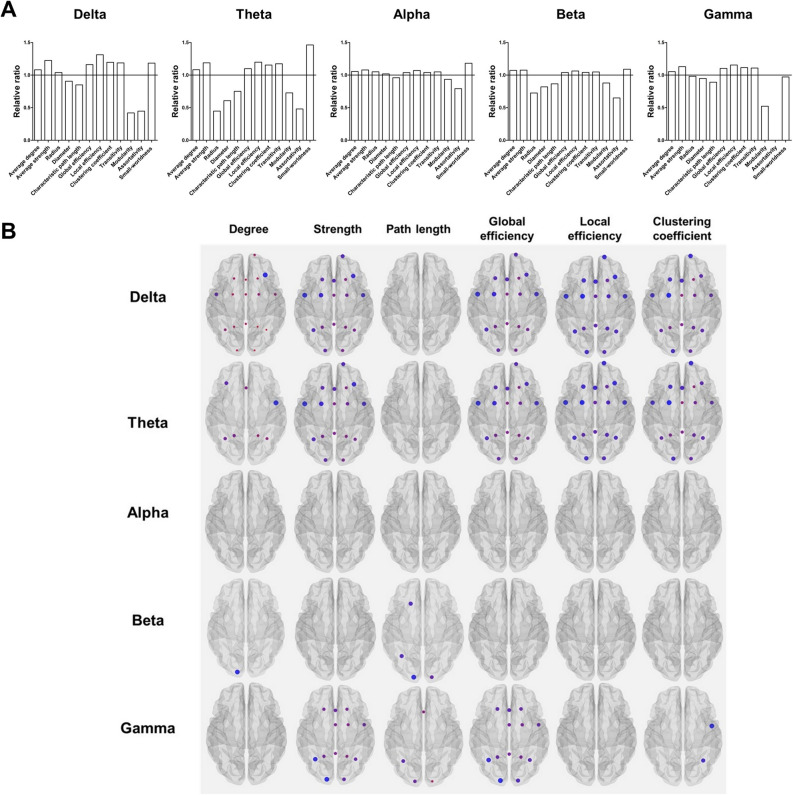
Results of graph-theoretic analyses and correlation analysis. (**A**) By setting the values of graph measures at baseline to 1.0 (shown as a line at the value of 1.0), the relative ratio of global graph measures after coffee consumption are presented. (**B**) Changes after coffee consumption in the nodal measures of graph-theoretic analyses are presented (FDR-corrected P < 0.05). Larger nodes indicate greater differences between the conditions. The regions showing higher significance of differences are colored red, whereas the regions showing lower significance of difference are colored blue.

Compared to baseline, the relative ratio of global graph measures after coffee consumption is presented in Fig. 5A. Average degree (except in gamma band), average strength (except in beta band), global efficiency (except in alpha and beta bands), and local efficiency (except in beta band) increased after coffee consumption relative to baseline in most frequency bands (FDR-corrected P < 0.05). The clustering coefficient in delta and theta bands, as well as transitivity in the delta, theta, and gamma bands, increased after coffee consumption relative to baseline (FDR-corrected P < 0.05). Compared to baseline, modularity in the delta, theta, and gamma bands decreased after coffee consumption (FDR-corrected P < 0.05). Significant differences in the nodal measures (degree, strength, global efficiency, local efficiency, and clustering coefficient) between the conditions were prominent mainly in the fronto-centro-parietal regions, especially in the delta and theta bands (Fig. 5B).

### Correlation analyses

**Figure 6:**
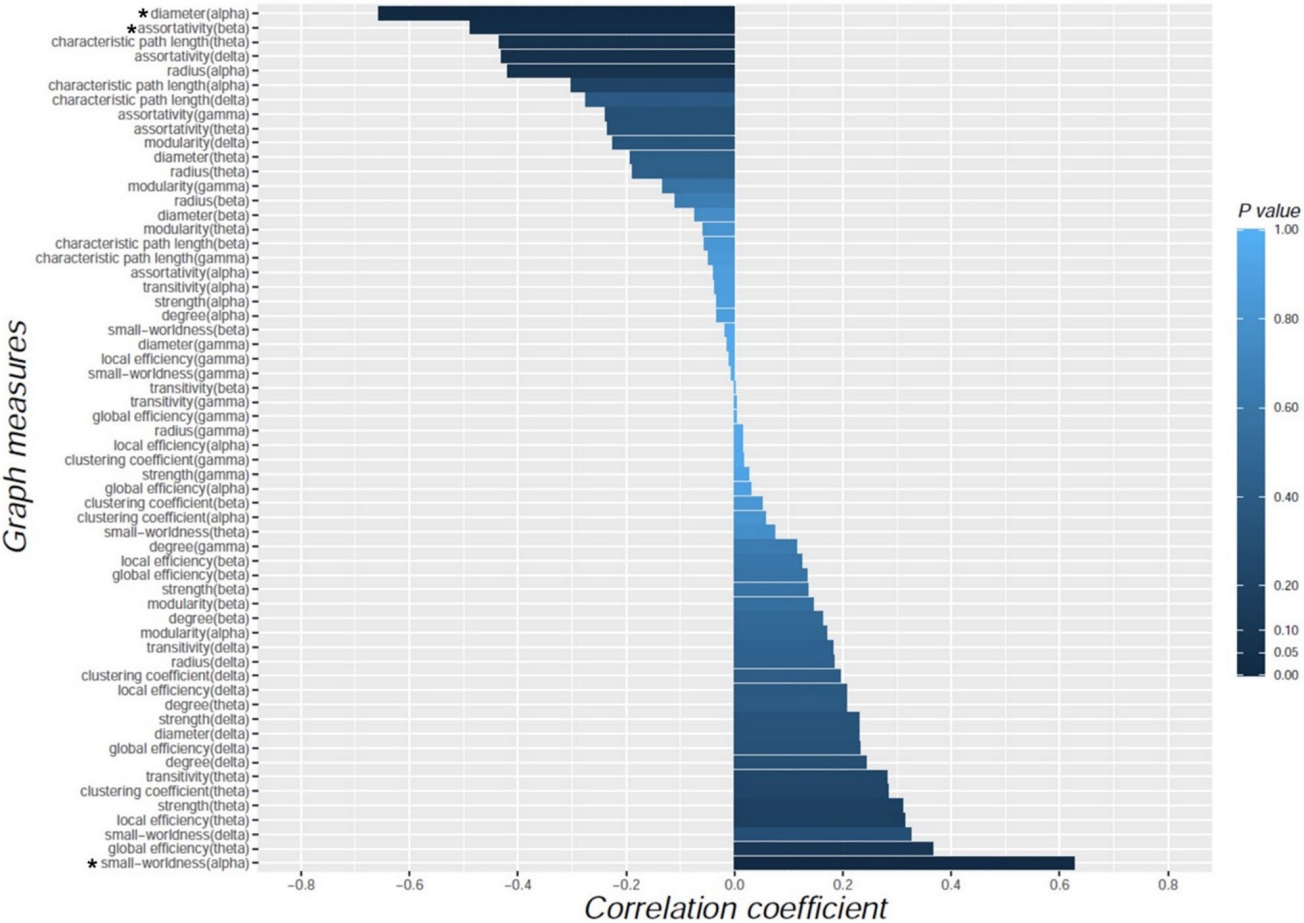
Correlations between the changes in global graph measures and the degree of improved performance on Trail Making Test Part B after coffee consumption. The horizontal bars represent the values of correlation coefficient. The color bar represents statistical significance (P value). Asterisks (*) represent statistical significance (P < 0.05).

The degree of improved performance in Trail Making Test Part B after coffee consumption was negatively correlated with diameter in the alpha band (r = ‒ 0.657, P = 0.002) and assortativity in the beta band (r = ‒ 0.488, P = 0.029), whereas it was positively correlated with small-worldness in the alpha band (r = 0.627, P = 0.004; Fig. 6). There was no relationship between the results of other neuropsychological tests and changes in graph measures.

## Discussion

We investigated the acute effects of caffeine on neurocognition and EEG FC in healthy adults. The major findings were as follows: (1) the property of EEG FC was reorganized toward a more efficient network after coffee consumption relative to baseline, (2) Performance in the Digit Span tests and Trail Making Test Part B was improved after coffee consumption, and (3) improved performance in the Trail Making Test Part B after coffee consumption was correlated with changes in graph measures reflecting a shift toward efficient network property.

The human brain is considered to be a large-scale complex network and has properties of efficient small-world networks that refer to locally well-connected clusters and efficient global connections^24,27^. The properties of small-world networks are known to enable higher rates of information processing and learning with a lower cost than those of random networks^28^. In terms of these network properties, changes in cognitive functional status or cognitive capacity might be associated with changes in the configuration of brain functional networks^27^. Indeed, there are several lines of evidence suggesting that loss of the small-world configuration might be implicated in the cognitive deficits observed in various brain disorders, such as Alzheimer’s disease, schizophrenia, and brain tumors^29–31^. Based on the aforementioned notion, our findings of changes in graph measures to high clustering and short path length after coffee consumption suggest that functional reorganization toward more efficient network properties might be a mechanism underlying the enhancement of cognitive function observed after coffee consumption.

The mechanism underlying the shift in FC toward efficient network properties after coffee consumption remains to be determined. It is believed that caffeine’s effect on cognition is associated with the blockade of the inhibitory properties of endogenous adenosine (particularly at A1 and A2A receptors), resulting in increased dopamine, norepinephrine, and glutamate release^4^. In addition, the cardiostimulatory effects of caffeine are considered to result from interactions with both adenosine and phosphodiesterase^32^. The caffeine-induced increases in dopamine and glutamate concentrations, coupled with phosphodiesterase inhibition, could be considered as a crucial mechanism underlying the net increase in the central nervous system and cardiovascular activity. Based on the actions of caffeine, it is plausible that the stimulatory effects of caffeine might directly lead to the reorganization of network properties toward a state of increased efficiency. A recent fMRI study showed that habitual coffee drinkers had distinct brain FC properties from non-coffee drinkers, which could support our speculation^33^. Further studies are needed to unveil the mechanisms underlying the changes in network properties after coffee consumption.

Our findings of improved performance in the Digit Span Forward test suggest that attentional function could be enhanced by coffee consumption, which is in line with previous observations that coffee consumption has beneficial effects on attention^7,34–36^. In addition, our findings of greater performance in the Digit Span Backward test^18^ after coffee consumption may support findings from previous studies that have shown the role of coffee in improving working memory^37–39^. A recent functional magnetic resonance imaging (fMRI) study found that the alerting network, known as being responsible for maintaining an alert state throughout task performance, recruited a distributed network of brain regions, primarily the thalamus and bilateral fronto-parietal regions^40,41^. Based on these fMRI findings, our results that FC changes after coffee consumption are mainly observed in the fronto-centro-parietal regions imply that improvement of attentional function might be derived from activation of the alerting network.

We also found that performance in the Trail Making Test Part B was improved after coffee consumption and that the degree of improvement of the test was correlated with the changes in graph measures reflecting a shift toward more efficient network properties. It is well known that the Trail Making Test Part B is a representative tool for evaluating the ability of executive function responsible for psychomotor speed, visuospatial searching, target-directed motor tracking, and set-shifting^42^. Therefore, our findings further support previous studies that showed the beneficial effects of caffeine on executive function and psychomotor speed^4,5,43^. Performance of executive controls requires activation of widespread prefrontal regions in concert with the anterior cingulate cortex^4,44,45^. These brain areas have been shown to be upregulated by caffeine^39,46^, supporting the stimulatory effects of caffeine on executive function. Moreover, dopamine was found to be a critical neurotransmitter for supporting executive function in these areas^47^. Given that dopamine concentrations can be increased by caffeine through blockade of the inhibitory properties of adenosine, caffeine may enhance executive function through the interaction of dopaminergic pathways with anterior cingulate and prefrontal cortical regions.

Our findings of the relationship between improved executive function and graph measures suggest that changing network topology toward more efficient network properties might be a crucial mechanism underlying the beneficial effects of coffee on executive function. Our speculation is supported by prior studies using fMRI that found increases in FC in multiple brain regions during the performance of the Trail Making Test Part B^47–51^. In addition, the aforementioned relationships were mainly observed in the alpha band, which is in accordance with a recent study showing that executive functions have a positive relationship with alpha coherence between regions of the right and left hemispheres^52^. Taken together, our findings support those of previous studies that coffee may enhance the FC responsible for performance on executive function, especially in the alpha band. Meanwhile, we did not find any changes in nodal graph measures after coffee consumption in the alpha band. The changes in global network properties without any region-specific changes in the alpha band suggest that coffee consumption might further enhance the improvement of physiological network efficiency responsible for activating cognitive function across the whole brain, rather than causing changes in the network properties of specific localized areas. Given the involvement of the dopaminergic pathways in executive function^47^, another plausible explanation is that our findings of changes in cortico-cortical network properties may not fully reflect the interactions of subcortical dopaminergic pathways with the cortical areas responsible for executive function.

We did not find any relationship between performance in Digit Span tests and graph measures. It is not fully understood why the results of the Digit Span tests, which reflect the function of attention/working memory, were not correlated with graph measures. However, it is plausible that there was a ceiling effect in the performance of the Digit Span tests in our cognitively normal population. In addition, given that attention/working memory is associated with not only cortical function but also various subcortical neurotransmitter systems (e.g., basal forebrain cholinergic systems and dopaminergic systems), a FC analysis evaluating the cortico-cortical network using EEG might not be sufficient to reveal the mechanism underlying the attention/working memory function.

There are several limitations of the present study that should be considered when interpreting our results. First, our study population was relatively small, and was only composed of highly educated young adults. Therefore, our results could not be generalized to the overall population, especially to the elderly. Second, we did not measure individual differences in biological susceptibility to caffeine or expectancy for coffee drinking to stimulate cognitive function^12^. Further studies incorporating measurements of caffeine blood level and investigation of a subjective expectation of coffee drinking as a cognitive enhancer may clarify the dose–response relationship and main contributor of the FC changes. Third, the results of the neuropsychological tests after coffee consumption may be biased due to learning effects. However, learning effects were likely mitigated by the use of different sets of contents in the repetition of the same tests. Finally, since canned coffee contains various ingredients other than caffeine, it is unclear whether our results were due to the effect of caffeine or the combined effects with other ingredients. Nevertheless, our study is the first EEG network analysis investigating the effects of canned coffee, containing a precisely controlled content of caffeine, on neurocognitive function.

The strength of our study is that FC was evaluated using two methods, coherence and PLI, which were compared to mitigate the limitations of scalp-level EEG analysis. We used two representative building blocks for characterizing brain FC in sensor space, coherence, and PLI, and obtained consistent results. Coherence is the most common method used to quantify the correlation between signals from different brain regions in terms of both amplitude and phase. In contrast, PLI measures the stability of the phase differences of short- and long-range neuronal activities over time independent of the amplitude of oscillations. This method is designed to reliably estimate phase synchronization against the presence of common sources such as volume conduction and active reference electrodes. In brief, it can be accomplished by discarding 0 and π phase differences between two time series^22^.

While not reported with the results, we performed classification of functional connectivities before and after coffee consumption using machine learning/deep learning algorithms. Eight global graph-theoretic measures of the PLI networks were used for classification using 70% and 30% training-test data split. We used supervised machine learning methods including support vector machine (SVM), k-nearest neighbor (kNN), decision tree, naïve Bayes, linear discriminant analysis (LDA), and logistic regression. Among the algorithms tested, the kNN (k = 4) exhibited the highest classification accuracy of 63.7%. This limited accuracy obtained may be because those machine learning techniques do not properly reflect geometric information based on channel locations. Observing the changes in functional connectivity between specific channels (Figs. 2 and 3) may be more informative for assessing the effects of drinking coffee. We note that several methods have been used to distinguish the EEG of patients with depression from control^53–56^. In particular, some methods had been proposed that detect depression with good accuracy using three-electrode EEG devices^54,55^. This suggests that performing channel selection with methods such as kernel-target alignment^56^ may provide additional insights into coffee consumption by identifying the key channels. We leave this to a future study.

## Conclusion

Our results support the general belief and previous notion that coffee improves cognitive function. Moreover, our findings suggest that the beneficial effects of coffee might be attributed to reorganization of FC toward more efficient network properties. Our findings of changes in network properties may provide novel insights into the biological mechanisms underlying the beneficial effects of coffee on cognitive function. Furthermore, the patterns of network reorganization could be quantitative markers for elucidating the mechanisms underlying the beneficial effects of coffee on cognition, especially executive function.
